# Allgrove syndrome with early neurodegeneration in a child: A case report from Syria

**DOI:** 10.1097/MD.0000000000047238

**Published:** 2026-01-16

**Authors:** Hadi Salame, Rida Jaber, Hussein Taher, Mohamad Olleik, Ali Farhat, Lina Khouri

**Affiliations:** a Faculty of Medicine, Damascus University, Damascus, Syrian Arab Republic; b Faculty of Medicine, AL-Sham Private University, Damascus, Syrian Arab Republic; c Damascus University, Children’s University Hospital, Damascus, Syrian Arab Republic.

**Keywords:** achalasia, adrenal insufficiency, alacrima, Allgrove syndrome, case report, neurodegeneration

## Abstract

**Introduction:**

Allgrove syndrome (AS), or “Triple A” syndrome, is a rare autosomal recessive disorder first described in 1978. It affects approximately 1 in a million individuals and is caused by mutations in the *AAAS* gene on chromosome 12q13. This gene encodes the ALADIN protein, essential for cellular function in various tissues. The syndrome is defined by a triad of clinical features: alacrima (absence of tears), achalasia (esophageal dysfunction), and adrenocorticotropic hormone-resistant adrenal insufficiency. Neurological involvement, including autonomic and peripheral neuropathies, is more commonly observed later in life, making early diagnosis challenging.

**Case presentation:**

A 4-year-old girl presented with vomiting, dysphagia, generalized weakness, alacrima, and skin hyperpigmentation. Addison disease was confirmed by elevated adrenocorticotropic hormone levels, and achalasia was diagnosed via a barium swallow test showing a bird’s beak sign. Although treatment was initiated, surgery was initially delayed due to the patient’s condition. Later, she developed seizures and neurological deterioration. Magnetic resonance imaging revealed cerebral atrophy, confirming the diagnosis of AS with neurological involvement. Treatment included medications targeting adrenal insufficiency and symptom management; however, there was no significant improvement in neurological symptoms or oral intake. Surgical intervention with Heller myotomy and gastrostomy led to improved feeding.

**Conclusions:**

AS can lead to serious complications, including life-threatening adrenal crises if undiagnosed. Early identification of glucocorticoid deficiency is vital to prevent mortality and long-term morbidity. Timely recognition and a multidisciplinary approach are essential. Regular follow-ups are necessary to manage neurological progression and support normal development in affected children.

## 1. Introduction

Allgrove syndrome (AS), also known as “3 A” syndrome, is a rare autosomal recessive disorder that was first described by Allgrove and his colleagues in 1978.^[1]^ It constitutes one of the rare genetic diseases affecting approximately 1 in a million people in the general population, although this estimate might be lower than the actual rate due to underdiagnosis.^[2]^ The condition arises due to alterations in the AAAS gene located on chromosome 12q13. This gene encodes ALADIN, a nuclear envelope protein that is extensively present in different human tissues.^[3]^ The syndrome is characterized by alacrima (lack of tears), achalasia (difficulty swallowing), autonomous neuropathy, and adrenocorticotropic hormone (ACTH) insensitivity. The condition typically appears during childhood and can cause dysphagia, hypoglycemia, and hypotension due to adrenal insufficiency.^[4]^ Autonomic and peripheral neuropathies are more commonly observed in adults.^[5]^ The incidence of the syndrome is difficult to determine due to its varied presentation, but it is more common in individuals of Black, Native American, Arab, and Asian descent.^[6]^ We present an interesting case of AS with early neuropathy. The patient was diagnosed with alacrima, adrenal insufficiency, and achalasia at the age of 4, completing the syndrome trilogy. Additionally, this is a rare case in which our patient presented early with neurological symptoms, with cerebral atrophy documented on magnetic resonance imaging (MRI) upon further investigation.

## 2. Case presentation

A 4-year-old female, born without complications to consanguineous parents, presented to our department with complaints of recurrent episodes of vomiting, dysphagia, and general weakness. The parents also noticed growth failure, which was confirmed after comparing the patient’s anthropometric data with the Syrian national growth chart for female children and adolescents: her height measured 94 cm (−1.5 standard deviations), her weight 12 kg (−2.5 standard deviations), and her head circumference 51 cm (within normal limits). Fundoscopic examination during the first evaluation was unremarkable.

The parents reported that an older brother complained of alacrima, claudication, and recurrent vomiting. The current patient had difficulties with speech, interacting only through gestures rather than phrases or words, despite having normal psychomotor development. She was able to sit at 7 months and walk at 14 months, but language and social milestones remained delayed. Her parents also noted a lack of tears when she cried.

On physical examination, hyperpigmentation spots were found on the lips, gingiva, bilateral lower eyelids, abdomen, and both knees. These findings raised suspicion of Addison disease. Consequently, ACTH and morning cortisol tests were ordered, resulting in levels of 1974 pg/mL and 17 µg/dL, respectively (normal values are 7.2–63 pg/mL and 5–25 µg/dL respectively according to the laboratory references). With elevated ACTH levels, a diagnosis of Addison disease was confirmed, and the patient was started on 20 mg/kg/d IV.

**Figure 1.**
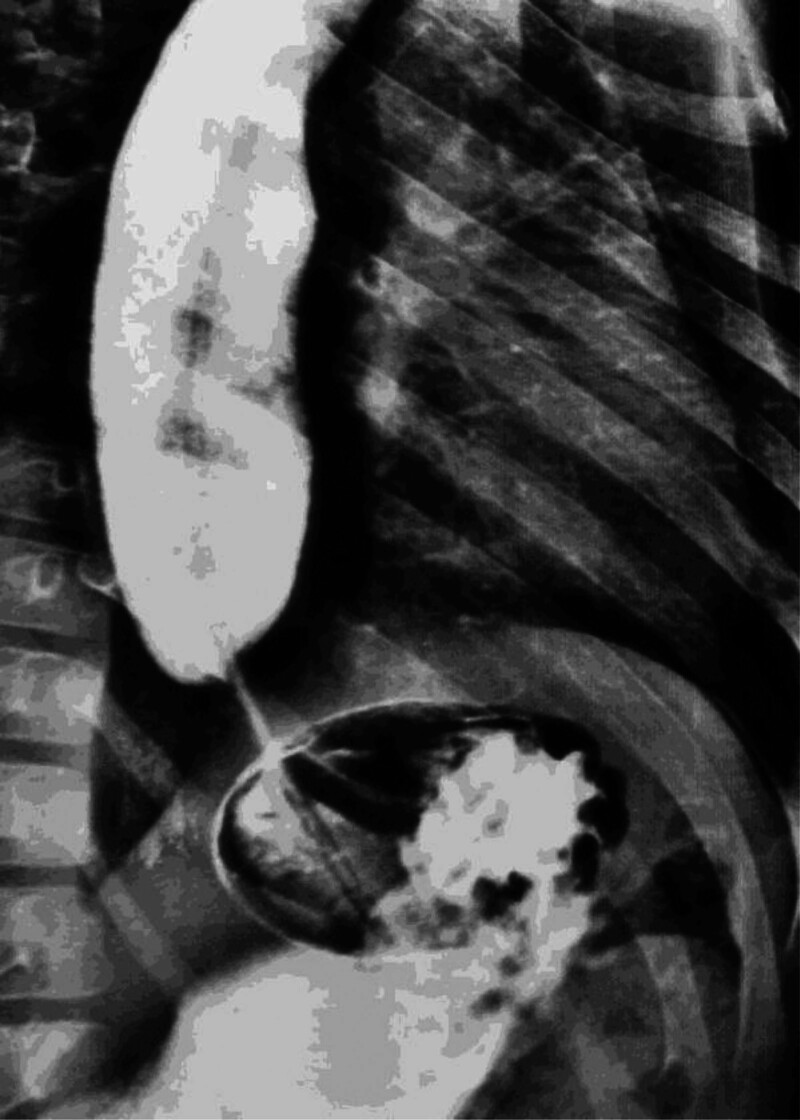
Barium swallow test showing narrowing of the lower esophageal sphincter as well as dilatation of the esophagus body (bird’s beak sign).

Further investigations, including a barium swallow test, revealed stenosis of the lower esophageal sphincter and complete body dilatation of the esophagus (bird beak sign), leading to a diagnosis of achalasia (Fig. 1). Treatment with nifedipine was initiated, and surgery was postponed until the child’s medical condition allowed her to undergo surgery. Subsequently, the patient was discharged. On follow-up, her ACTH laboratory value was normal (28 pg/mL).

**Figure 2.**
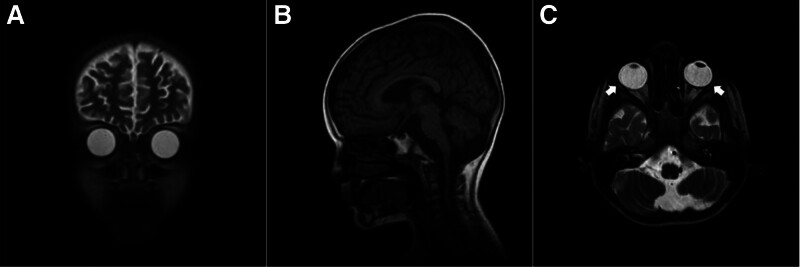
MRI revealing (A) cortical atrophy in the cerebrum, (B) cerebellar atrophy, thinning of the corpus callosum, mega cisterna magna, and (C) bilateral atrophy of lacrimal glands (arrows). MRI = magnetic resonance imaging.

**Figure 3.**
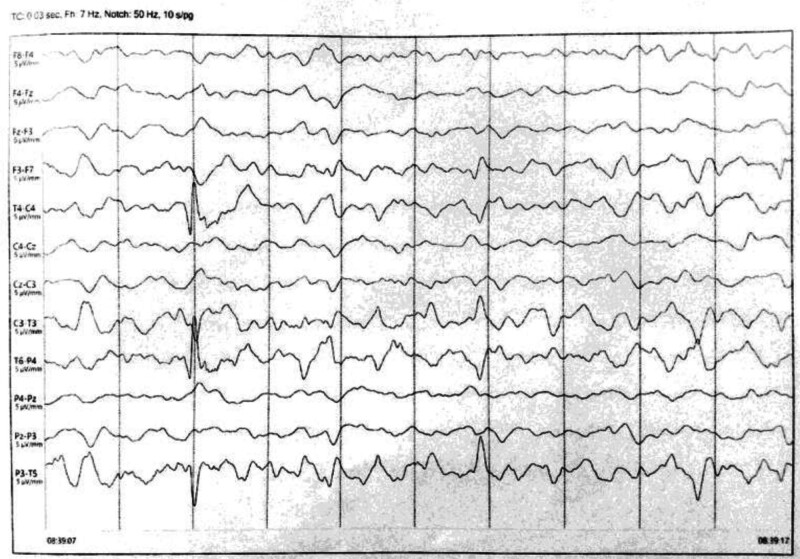
EEG with abnormal background and generalized discharges. EEG = electroencephalogram.

One month later, the patient started to experience fever followed by a seizure, resulting in admission to the intensive care unit where the blood glucose level measured 65 mg/dL. During the episode, the parents noticed frothing from the mouth and bilateral eye adduction. On the Glasgow Coma Scale, the girl scored 4/4 on eye opening, 5/6 on motor response, and 1/5 for the verbal response thus a total score of Glasgow Coma Scale was 9/15. She had an unconscious gaze and was unresponsive to speech. Random and involuntary movements of the head and limb were observed. Hypertonia of the lower extremities and hyperreflexia (+3 on both sides) were noted. Lumbar puncture revealed a normal cell count. MRI revealed cortical atrophy with high signal intensity in the periventricular white matter, cerebellar atrophy, thinning of the corpus callosum, and a mega cisterna magna. Furthermore, atrophy of the lacrimal glands on both sides was observed (Fig. 2). For additional evaluation, electroencephalography showed a consistent abnormal pattern with generalized discharges (Fig. 3).

Considering the triad of Addison disease, Achalasia, and Alacrima, along with neurological abnormalities, a final diagnosis of triple A syndrome was made upon the clear clinical findings. Genetic analysis could not be performed due to resource limitations in Syria.

Treatment with levetiracetam (20 mg/kg/d) was initiated for seizure control. Haloperidol (0.025 mg/kg/d) was added for its tranquilizing effects to reduce the intensity and frequency of involuntary movements; however, no improvements were observed. Poor nutritional status was noted, prompting surgical intervention for achalasia.

A Heller myotomy was performed on the anterior part of the esophageal wall, followed by fundoplication and medial narrowing of the right diaphragmatic crus to release pressure on the lower esophageal sphincter. A Stamm gastrostomy was also performed to allow direct feeding into the stomach. The patient was placed on a nothing per mouth regimen and a nasogastric tube until the gastrostomy tube completely drained.

After recovery from surgery, tolerance for oral liquids and solids improved. A higher dose of haloperidol (0.05 mg/kg/d) remarkably corrected the child’s movements. The child is now in stable condition and awaits full recovery before being discharged.

## 3. Discussion and conclusions

AS is defined by a triad of adrenal insufficiency (Addison disease), failure of the lower esophageal sphincter to relax (achalasia), and absence of tear secretion (alacrima). It is also recognized as a 3A syndrome, and it is proposed to be categorized as a 4A syndrome, with neurological symptoms such as autonomic dysfunction, motor neuropathy, sensory disorders, mental retardation, and other related neurological conditions being the fourth component.^[5]^ When both autonomic neuropathy and amyotrophy coexist, the term 5A syndrome is used.^[7]^ Our pediatric patient was diagnosed with achalasia, alacrima, and adrenal insufficiency, followed by seizures and neurological abnormalities, aligning with all the “A”s of the syndrome. Consequently, she was diagnosed with AS, which features an early manifestation of uncommon neuropathy.

The onset of different symptoms of AS varies with age. Alacrima typically presents in early infancy, while symptoms of achalasia may be reported as early as 6 months of age. Adrenal insufficiency is not present at birth but can develop at any age within the first 2 decades of life.^[8]^ Neurological symptoms usually emerge in the second decade of life and adulthood; however, in some cases, they may be among the presenting symptoms in childhood.^[9,10]^ In our patient, a lack of tears was noted by the patient’s parents since infancy. By the age of 4, adrenal insufficiency and achalasia were diagnosed, followed shortly by seizures and signs of neurodegeneration.

The majority of individuals exhibit typical signs of primary adrenal insufficiency, such as experiencing hypoglycemic seizures and shock. Children with recurrent vomiting, dysphagia, and failure to thrive are less likely to experience symptoms associated with crying without tears. Other presentations may include hyperpigmentation, developmental delay, nasal speech, or orthostatic hypotension.^[11]^ The diminished or lack of lacrimation that accompanies this condition often results in dehydration-induced keratopathy, which can be detected through rose Bengal staining.^[12]^ Patients with achalasia may also exhibit respiratory symptoms such as cough, aspiration, hoarseness, dyspnea, wheezing, or sore throat in as many as 40% of cases.^[13]^ In certain instances, occasional or recurrent pneumonias have raised concerns among the medical team, prompting additional investigations and subsequent diagnosis of 3A syndrome.^[14]^ Vomiting and dysphagia were the primary problems experienced by our patient, in addition to the absence of tears while crying and hyperpigmented areas in her body. Subsequently, she suffered from fever followed by seizures, announcing the onset of neurological problems.

Individuals diagnosed with AS often exhibit concurrent neurological abnormalities and may involve the central, peripheral, and autonomic nervous system. These manifestations typically appear at a later age than other symptoms.^[15]^ Previous studies have indicated that autonomic nervous system disorders can manifest in various ways, such as hypotension or hypertension, changes in sweating, cardiac arrhythmias, abnormal pupillary reflexes, and anisocoria.^[16]^ It may also include sensorimotor polyneuropathy, autonomic dysfunction, amyotrophy, dysarthria, ataxia, optic atrophy, and intellectual impairment. Some cases also report evidence of cognitive defects, pyramidal syndrome, distal muscular dystrophy, hyperreflexia, cerebellar dysfunction, dysautonomia, neuro-ophthalmological signs, bulbar and facial symptoms, and microcephaly.^[7]^ Furthermore, patients may present with delayed developmental milestones, hypotonia, gait abnormalities, and hearing deficits.^[17]^ Our patient exhibited hyperreflexia, lower limb hypertonia and spasms, an unconscious gaze, and involuntary head and limb movements. She also showed mild developmental delay, especially in language and communication, and abnormal gait, with stature-weight growth delay based on the Syrian national growth chart for female children and adolescents.

For the diagnosis of AS, investigations may include Schirmer test for alacrima, confirming the presence of reduced or absent tears, where wetting of filter paper < 10 mm indicates dry eye and wetting of > 5 mm indicates severe dry eye.^[18]^ An MRI scan of the orbit can be conducted to examine lacrimal gland atrophy.^[19]^ On the other hand, orbital CT scans reveal the absence or decrease of lacrimal glands and a depletion of secretory granules in acinar cells.^[18]^ The diagnosis of achalasia is clinically suspected based on symptoms, but it is important to note that upper gastrointestinal endoscopy can reveal esophageal dilation and the retention of food or secretions, while barium swallow can reveal a bird-beak appearance in the lower esophageal region. Adrenal insufficiency is strongly suggested by cortisol levels at 8 am, along with concomitant ACTH measurements. The expected findings in ACTH sensitivity are low cortisol combined with markedly high ACTH.^[20]^ Electroencephalograms, brain MRI, and evoked potential and nerve conduction studies are preferred for patients who present with seizures or other neurological symptoms.^[21]^ High ACTH levels were detected in the child, confirming adrenal insufficiency. Barium swallow was crucial in manifesting achalasia. For her neuropathy, electroencephalogram and MRI scans were conducted, revealing cortical atrophy, cerebellar atrophy, high signal intensity in the periventricular white matter, thinning of the corpus callosum, and the presence of a mega cisterna magna, in addition to bilateral atrophy of the lacrimal gland.

A cure for triple A syndrome is unfortunately unavailable; treatment primarily revolves around addressing specific signs and symptoms associated with the condition. The short-acting glucocorticoid hydrocortisone is the treatment of choice for patients with adrenal insufficiency; however, in some cases, fludrocortisone may also be required.^[22]^ Artificial tears are administered to the eyes to prevent keratopathy and corneal ulcers in case of alacrima.^[23]^ When treatment failure occurs, surgeons are more likely to prefer surgical treatment for achalasia correction.^[24]^ To reduce the risk of esophageal reflux after Heller myotomy, fundoplication is suggested.^[25]^ In our case, the patient received intravenous hydrocortisone in addition to nifedipine, omeprazole, and lubricant eye drops. Subsequently, Heller myotomy was performed to improve food intake, followed by treatment with haloperidol for involuntary movements.

AS is a rare multisystem disease that causes many complications. The leading cause of mortality is an undiagnosed adrenal crisis presenting as a hypoglycemic seizure early in the course of the disease.^[2]^ According to Zamanfar et al, alacrima is recognized as the initial clinical indication of triple A syndrome. Early detection of glucocorticoid deficiency is crucial for preventing hypoglycemic convulsions, neurological complications, and fatalities. Meticulous replacement of glucocorticoids is essential to avert adrenal crisis and support regular growth and development.^[26]^ Therefore, general practitioners and pediatricians should be able to recognize and diagnose a patient with AS as early as possible to prevent complications, especially neuronal complications, as in our case, to improve normal development. Regular follow-up is recommended to provide proper care and monitor patient progress.

## Author contributions

**Supervision:** Lina Khouri.

**Writing – original draft:** Hadi Salame, Rida Jaber, Hussein Taher, Mohamad Olleik, Ali Farhat.

**Writing – review & editing:** Hadi Salame, Rida Jaber, Hussein Taher, Mohamad Olleik, Ali Farhat.
